# MEK inhibition with trametinib is a successful therapy in ganglioglioma

**Published:** 2020-05-08

**Authors:** Eliza Baird Daniel, Douglas E Ney, Jean M Mulcahy Levy

**Affiliations:** 1 Department of Pediatrics, University of Colorado Denver, Aurora, USA; 2 The Morgan Adams Foundation Pediatric Brain Tumor Research Program, Children’s Hospital Colorado, Aurora, USA; 3 Departments of Neurology and Neurosurgery, University of Colorado School of Medicine, Aurora, USA

**Keywords:** ganglioglioma, MEK inhibitor, Trametinib, BRAF, low-grade-glioma

## Abstract

Gangliogliomas are predominantly low-grade primary brain tumors comprised of neuronal and glial components that are found in both pediatric and young adult populations. In the majority of cases, surgical resection of these tumors is curative. However, tumor location in eloquent centers of the brain can make surgical intervention inappropriate. Additionally, a subset of tumors progress to anaplastic ganglioglioma which carries a poor prognosis, despite resection. Activating mutations in the MAPK pathway, such as BRAF V600E, have been identified in many of these tumors. Tumors carrying such mutations have demonstrated susceptibility to MEK inhibition therapy. However, there remains a subset of ganglioglioma that do not contain a known mutation in the MAPK pathway and thus have not been targeted with MEK inhibition therapy. Here, we present a young adult ganglioglioma patient without identified MAPK pathway activation mutations who demonstrated a significant and sustained response to MEK inhibition with trametinib.

## Introduction

Gangliogliomas are primary brain tumors most commonly arising in children and young adults less than 30 years old but are seen in the older adult population as well. Like pediatric patients, adult ganglioglioma are predominantly low-grade (91.4%) [1]. While they are generally associated with a favorable prognosis following gross total resection (GTR), a subset may progress to anaplastic ganglioglioma while other tumors are in eloquent centers of the brain and unable to be resected [2]. In adults it has been reported that only 59% of patients are able to achieve a GTR which can have an adverse effect on outcomes [1]. Ganglioglioma patients have been reported to have progression free survival (PFS) of 58% at 5 years and only 37% at 10 years with extent of resection most strongly associated with PFS. Reported medium time to progression was only 1.8 years with a subtotal resection (STR) while this extended to 16.7 years with a GTR [3]. The role of chemotherapy and radiation for patients with STR is still under debate and maximal safe resection remains the gold-standard therapy [1].

In recent years there has been extensive work describing the genetic landscape and potential therapeutic targets in these tumors. The BRAF V600E mutation makes up a significant amount of the genetic alterations found in ganglioglioma, but other MAPK and non-MAPK pathway mutations have been described [4]. Pekmezci et al. note that in the majority of cases, ganglioglioma showed BRAF alterations however, in tumors lacking identified BRAF mutations, 69% demonstrated mutations that are predicted to activate the MAPK pathway such as alterations in KRAS, FGFR2, NF1, and RAF1 [4].

Given the prevalence of MAPK activation described in gangliogliomas, MEK inhibitors such as trametinib may provide a significant therapeutic option. MEK inhibition in conjunction with BRAF inhibition has shown promising results in BRAF V600E mutation in adult anaplastic ganglioglioma [2]. Additionally, trametinib therapy in other low-grade gliomas with known activation mutations in the MAPK/ERK pathway shows promising response rates and regimen tolerance in pediatric patients [5]. Ultimately, a subset (estimated to make up 10–60% of ganglioglioma) containing BRAF V600E/ MAPK mutations respond to MEK inhibition however effects of this treatment on other subsets harboring genetic alterations both within and outside of the MAPK pathway remains largely unknown [4]. We present an adult GG patient without identified MAPK pathway activation mutations who demonstrated a significant and sustained response to MEK inhibition with trametinib

## Case Report

**Figure 1.**
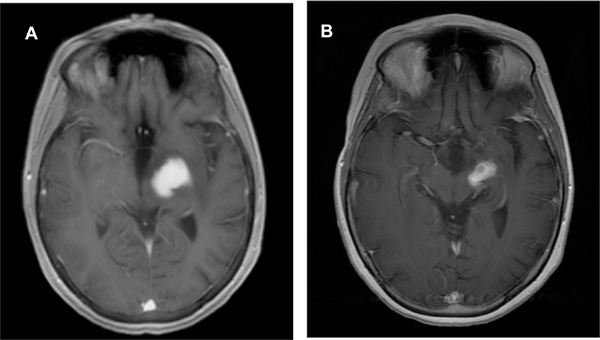
Ganglioglioma without identified MAPK pathway activation mutations demonstrates a significant and sustained response to MEK inhibition with trametinib **T1-weighted post-contrast axial images:** (A) showing contrast enhancing lesion centered in the left thalamic region. (B) Follow-up imaging 6 months post-initiation of trametinib demonstrated a significant partial radiographic response

A 32-year-old woman presented with two to three years of progressive right-sided arm greater than leg weakness. Her history consisted of a bifrontal brain mass at the age of 11 years. At that time, she underwent biopsy, which was said to be consistent with germinoma, however, the pathology was not able to be re-reviewed. Adjuvant treatment consisted of craniospinal radiotherapy with resolution of disease without recurrence. At the time of her current presentation, MRI imaging showed an enhancing left thalamic mass (Figure 1a). Biopsy was undertaken which was consistent with a WHO grade 1 ganglioglioma, negative for BRAF or other targetable mutations. Due to the location of the lesion she was not a candidate for attempt at GTR. She initiated treatment with trametinib 2 mg daily which was complicated by diffuse rash and photosensitivity which resolved after drug interruption to allow rash recovery and dose reduction to 1.5 mg daily. Follow-up at 6 months showed significant radiographic improvement (Figure 1b) as well as improvement in strength. Clinical and radiographic response persists at 18 months post-initiation of trametinib and she continues on 1.5 mg daily.

## Discussion

Due to the great majority of characterized mutations in ganglioglioma identified as players in the MAPK pathway [4], we hypothesized for this patient that ganglioglioma is largely a single pathway (MAPK) driven neoplasm. Thus, we predicted that tumors without an identified mutation within the MAPK pathway, such as this patient’s tumor, would also be responsive to MEK inhibition. The success of trametinib in this patient highlights the potential benefit of this class of drugs for patients with unresectable ganglioglioma.

There are multiple ongoing studies of MEK inhibitors for central nervous system tumors in pediatric patients including the Pediatric MATCH (NCT03213691) and other trials (NCT02285439, NCT01089101). And while there are several studies including targeting the MAPK pathway in brain metastatic melanoma and other tumors, there are currently no primary adult CNS tumor studies evaluating the potential of MEK inhibitors for patients with verified or presumed MAPK pathway activation. This case and the success of MEK inhibition in primary pediatric low-grade gliomas would suggest a potential avenue of new research for adult low-grade and ganglioglioma patients [5].
